# Effects of air pollution on myopia: an update on clinical evidence and biological mechanisms

**DOI:** 10.1007/s11356-022-22764-9

**Published:** 2022-08-29

**Authors:** Tianyi Yuan, Haidong Zou

**Affiliations:** 1 Department of Ophthalmology, Shanghai General Hospital, School of Medicine, Shanghai Jiao Tong University, Shanghai, China; 2 Shanghai Eye Diseases Prevention & Treatment Center, Shanghai Eye Hospital, Shanghai, China; 3 National Clinical Research Center for Eye Diseases, Shanghai, China; 4 Shanghai Engineering Center for Precise Diagnosis and Treatment of Eye Diseases, Shanghai, China

**Keywords:** Myopia, Air pollution, Peripheral hyperopia defocus, Dopamine, Retinal ischemia

## Abstract

Myopia is one of the most common forms of refractive eye disease and considered as a worldwide pandemic experienced by half of the global population by 2050. During the past several decades, myopia has become a leading cause of visual impairment, whereas several factors are believed to be associated with its occurrence and development. In terms of environmental factors, air pollution has gained more attention in recent years, as exposure to ambient air pollution seems to increase peripheral hyperopia defocus, affect the dopamine pathways, and cause retinal ischemia. In this review, we highlight epidemiological evidence and potential biological mechanisms that may link exposure to air pollutants to myopia. A thorough understanding of these mechanisms is a key for establishing and implementing targeting strategies. Regulatory efforts to control air pollution through effective policies and limit individual exposure to preventable risks are required in reducing this global public health burden.

## Introduction

**Fig. 1:**
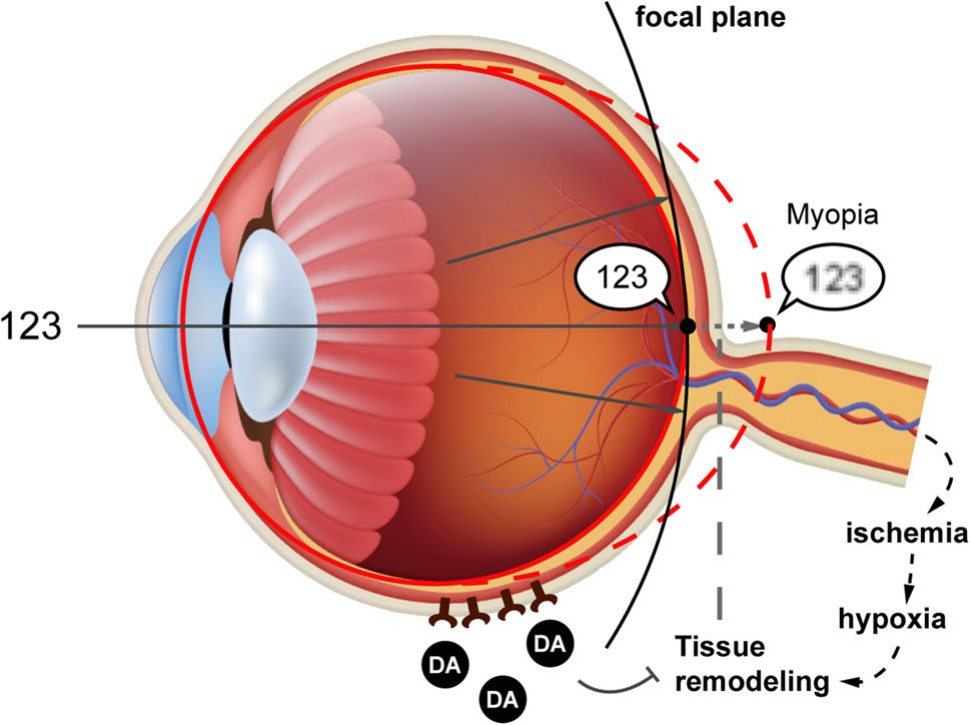
The underlying pathways of myopia pathogenesis. The clear “123” indicates the vision of emmetropia, while the blurred one represents myopia. When the peripheral hyperopia defocus occurs, the incoming light rays are focused behind the retina surface and axial growth is lengthened owing to the tissue remodeling of the sclera. The axial elongation can also be promoted by the hypoxia marker upregulated by retinal ischemia, while inhibited by the release of retinal dopamine. *DA* dopamine

Myopia (short-sightedness or near-sightedness) is defined as a spherical equivalent (SE) ≤  − 0.5diopters (D)(Baird et al. 2020). The World Health Organization (WHO) recognizes that individuals with myopia are at a substantially increased risk of potentially blinding myopic pathologies that are not prevented by optical correction (Morgan et al. 2012). The pathologic complications include myopic macular degeneration, choroidal neovascularization, cataract, and glaucoma (Pan et al. 2012). For more than one third of adults, myopia can progress during the third decade of life (Lee et al. 2022). The continuous progression of myopia may result in significant reductions in work, educational productivity, and overall quality of life. However, approximately 2 billion individuals (28.3% of the global population) are diagnosed with myopia worldwide, and its prevalence is estimated to increase to 4.76 billion individuals (49.8% of the global population) by 2050 (Baird et al. 2020; Morgan et al. 2018). The cumulative incidence of myopia among Chinese school-aged children and adolescents has increased consistently, reaching 91.3% (minimum to maximum: 83.7–96.7%) upon graduation from high school (Chen et al. 2021). The causes of this pandemic remain unclear. The commonly agreed underlying mechanisms are peripheral hyperopia defocus, dopamine (DA) pathway, and retinal ischemia (Fig. 1). Such mechanisms are affected by air pollutants and may potentially link myopia to air pollution.

Air pollution is a complex mixture of gaseous components and particulate matter (PM) suspended in air (Miyazaki et al. 2020). Gaseous pollutants include carbon monoxide (CO), nitrogen oxides (NO_x_), and ozone (O_3_) (Ruan et al. 2019). Ambient PMs are emitted by the combustion of fossil fuels or natural sources, such as volcanic eruptions and wildfires (Miyazaki et al. 2020). PMs are categorized by their diameter: particulate matter 10 (PM_10_), particulate matter 2.5 (PM_2.5_), and ultrafine PMs (Miyazaki et al. 2020; Gao et al. 2019). A report from the Organization for Economic Co-operation and Development indicated that outdoor air pollution could cost $2.6 trillion a year, by 2060 (Bai et al. 2018). According to the WHO, approximately seven million individuals die from air pollution annually (Areal et al. 2022). There are regions of the world, notably in Asia, that bear the greatest disease burden from air pollution (The 2017). Exposure to air pollution has been associated with asthma, cognitive functioning, neurodegenerative diseases, dry eye disease, blepharitis, conjunctivitis, and cataracts (Orru et al. 2017; Schraufnagel et al. 2019). In fact, many epidemiological evidences have proved greater air pollutant concentrations to be strongly associated with an increased likelihood of myopia (Yang et al. 2021; Ruan et al. 2019; Wei et al. 2019; Dadvand et al. 2017).

In this review, we summarize the currently available data on the association between air pollution and myopia for the lack of a comprehensive review on this topic so far. We aim to explore the underlying pathophysiological mechanisms hoping to inspire future research and provide more insights into the clean-air regulation efforts for governments.

## Epidemiological evidence of ambient air pollutant exposure on myopia

Several air pollutants are positively correlated with the incidence of myopia (Ruan et al. 2019; Wei et al. 2019; Dadvand et al. 2017; Yang et al. 2021), as shown in Table 1. PM_2.5_, particles ≤ 2.5 µm in aerodynamic diameter, are primarily produced by motor vehicles, power plants, and other combustion sources (Adar et al. 2010). The small size of PM_2.5_ makes them particularly deleterious to health, causing respiratory and cardiovascular morbidity. Wei et al. (2019) conducted a retrospective cohort study including 97,306 children aged 6–12 years and found that those who were exposed to higher PM_2.5_ concentrations had a higher cumulative incidence of myopia. A cohort study of 61,995 children in 7 Chinese provinces/municipalities reported the associations of PMs between prevalent visual impairment and visual acuity levels (Yang et al. 2021). Similar observations made by Dadvand et al. (2017) suggested that there was an increase in the likelihood of myopia (as surrogated by spectacle use) associated with exposure to residential PM_2.5_ absorbance. To confirm the relationship between PM_2.5_ and myopia, Wei et al. (2019) treated 3-week-old hamsters with 100 μg/mL PM_2.5_ twice a day. After 3 weeks, the hamsters developed myopia (change in refractive error =  − 1.513 ± 2.092 D), whereas the hamsters in the control and PM_2.5_ + resveratrol groups did not (change in refractive error = 0.913 ± 1.772 D and 0.65 ± 1.36 D, respectively).

**Table 1:** Summary of clinical studies on the effects of ambient air pollutant exposure on myopia

Study	Type of study	Study period and location	Sample size	Age (years)	Environmental exposure measured	Outcome measured	Findings
Wei et al. (2019)	Retrospective cohort study	2000–2012, Taiwan, China	97,306 children	6–12	Daily average concentrations of PM_2.5_ and NO_x_ calculated from the air quality-monitoring data	Myopia mentioned at least two times by an ophthalmologist	The incidence rate of myopia increased with exposure to PM_2.5_ and NO_x_ from 15.8 to 24.5 and from 13.7 to 34.4 per 1000 person-years
Ruan et al. (2019)	Cross-sectional survey	2007–2010, South Africa, Ghana, Mexico, China, India, and the Russian Federation	33,626 adults	≥ 50	Annual concentrations of fine PM_2.5_ and O_3_ estimated with the satellite data and chemical transport model	At least one of the following three standards: (1) have been diagnosed as myopia by a medical professional; (2) have a good near vision but distant objects appear blurred; (3) must wear contact lenses or eyeglasses to see distant object	*J*-shaped associations between PM_2.5_ and O_3_ concentrations with myopia The adjusted prevalence ratio identified as 1.12 and 1.26 for each standard deviation increase in PM_2.5_ and O_3_ concentrations above their threshold
Dadvand et al. (2017)	Cross-sectional analyses	2012–2015, Barcelona, Spain	2727 schoolchildren	7–10	NO_2_ and PM_2.5_ light absorbance at home predicted by land-use regression models NO_2_ and black carbon at school light absorbance measured by monitoring campaigns	The use of spectacles	An IQR increase in NO_2_ level at home and school associated with 16% and 32% increase in spectacle use
	Longitudinal analyses		1812 schoolchildren				The residential exposure to PM_2.5_ absorbance and school exposure to BC absorbance getting stronger compared to those of cross-sectional analyses
Yang et al. (2021)	Cross-sectional study	2013, 7 provinces or municipalities in China	61,995 children	6–18	PM_2.5_, PM_10_, and NO_2_ estimated at a 0.1° × 0.1° resolution using machine learning methods	Visual impairment defined the unaided distance visual acuity lower than or equal to 4.9 (logarithm of the minimum angle of resolution 0.10 or Snellen 5/6 equivalent) in the worse eye	The IQR increase in PM_2.5_, PM_10_, and NO_2_ associated with a 1.267-, 1.142-, and 1.276-fold increased odds of visual impairment

*PM*_*2.5*_ particulate matter 2.5, *PM*_*10*_ particulate matter 10, *NO*_*x*_ nitrogen oxides, *O*_*3*_ ozone, *NO*_*2*_ nitrogen dioxide, *BC* black carbon, *IQR* interquartile range.

NO_x_ is a collective expression of nitric oxide (NO) and nitrogen dioxide (NO_2_), which transform into each other in the atmosphere. They are primarily emitted from power stations, motor vehicles, and other industrial combustion processes (Gao et al. 2022). Yang et al. reported that long-term exposure to NO_2_ was associated with increased odds of visual impairment (Yang et al. 2021). The cross-sectional analyses conducted by Dadvand et al. (2017) observed that an increase in NO_2_ levels at home and school was associated with an increase in spectacle use and a higher risk of myopia. Similar conclusions were drawn for NO_x_ concentrations (Wei et al. 2019).

Ground-level O_3_ is a typical secondary pollutant produced by photochemical reactions and is a potent greenhouse gas with both direct and indirect effects on human health (Luo et al. 2021; Xu et al. 2022; Montes et al. 2022). Ruan et al. (2019) discovered a synergistic interaction of two air pollutants on myopia, and the joint effect of high PM_2.5_ and high O_3_ on myopia (95% confidence interval (CI): 1.23, 1.73) was greater than the sum of their individual effects with a synergistic index of 1.81 (95% CI: 0.92, 4.94).

Although current studies are limited and include different air pollutants, they have all revealed a strong correlation between air pollution and myopia.

## Potential biological mechanisms of air pollutants on myopia

Eyes are exposed to ambient air pollution, making them prime vulnerable targets for the adverse effects of such exposure. O_3_ is linked to the overexpression of conjunctival interleukin (IL)-6 and tumor necrosis factor (TNF)-α (Jung et al. 2018), which are two typical inflammatory cytokines that initiate ocular inflammation. PM and NO_x_ can stimulate the formation of reactive oxygen species (ROS) and free radicals, which lead to oxidative stress (Lasagni Vitar et al. 2019; Wei et al. 2019). Prolonged elevated levels of ROS can result in redox disruption of metabolic, signaling, and transcription processes, which cause oxidative damage to macromolecules in both the cornea and retina (Lasagni Vitar et al. 2019; Liu et al. 2020; Ying et al. 2021).

In this case, the potential pathophysiology of air pollutants on myopia is classified into direct and indirect ways. The direct pathway refers to the direct distribution and concentration of pollutants on the eye and airway, leading to enhanced inflammation which is closely related to peripheral hyperopic scatter and retinal ischemia. The indirect one refers to the decreased release of DA in the eyes caused by less outdoor light and more air pollution.

### Indirect pathway: the DA pathway

DA is a neurotransmitter used by a class of amacrine cells that plays an important role in the retina, mediating eye functions such as visual signaling, ocular development, and refractive adjustment (Zhang and Deng 2020; Norton 2016; Zhou et al. 2017). Previous experiments in multiple species have suggested that DA acts as a “stop” signal in progression of myopia. Animal models, including chicks, mice, and primates, have demonstrated that dopaminergic compound administration can retard ocular growth by slowing vitreal chamber elongation and significantly inhibit the development of myopia in a similar dose-dependent manner (Thomson et al. 2020b, 2020a). In mice, this regulation may involve D_2_ receptors, a DA receptor subtype located in the retina (Huang et al. 2022; Thomson et al. 2020b). The release of retinal DA can participate in the retina-to-sclera signaling cascade, which induces scleral remodeling in response to sunlight stimuli (Grzybowski et al. 2020).

As the main component of sunlight, cumulative ultraviolet (UV) exposure can influence the presence of myopia (Kearney et al. 2019; Williams et al. 2017) through the DA pathways, particularly in adolescence and young adulthood (Williams et al. 2017). In rabbits, the abnormal elongation of the myopic eye was effectively controlled 1 month after ultraviolet A (UVA; a band of UV whose wavelengths range from 315 to 400 nm (Bajgar et al. 2021)) irradiation and almost halted 3 months after treatment (Rong et al. 2017). Similarly, posterior scleral cross-linking induced by riboflavin-UVA can slow the progression of myopia (Han et al. 2021; Lai et al. 2021; Dotan et al. 2016; Li et al. 2017). Serum metabolomic and lipidomic studies carried out by Du et al. (2020) revealed five pathways showing regulatory relationships with D_2_Rs in myopia: steroid biosynthesis, arginine and proline metabolism, linoleic acid metabolism, alpha-linolenic acid metabolism, and sphingolipid metabolism. Kato et al. (2019) suggested that UVA absorption had a direct effect on fibroblasts of the sclera and cornea. Consequently, solar UV radiation can be related to a change in layer thickness and/or rearrangement inside axons in the nerve fiber layer, as well as the connectivity between several different cell types in the retina to slow down myopic eye growth (Landis et al. 2021; Wirz-Justice et al. 2021; Lingham et al. 2020; Swiatczak et al. 2019).

The reduction in UV exposure that reaches the Earth’s surface occurs during the synthesis and emission of air pollutants (Borysov et al. 2020; Kalluri et al. 2021; Manisalidis et al. 2020, United Nations Environment Programme 2016). It is acknowledged that PM_2.5_ in the atmosphere can directly reflect solar radiation back into space because the physical properties of particles are strongly curved interfaces (Riva et al. 2021). Black carbon (BC) emitted by wildfires across the globe can persist in the atmosphere for days to weeks owing to its highly adhesive surface (Borysov et al. 2020). Such persistent increases in carbonaceous aerosols would significantly reduce UV radiation by chemical processes, such as oxidation and light-catalyzed reaction (Manisalidis et al. 2020; Guo et al. 2021; United Nations Environment Programme 2016). Owing to the impact of air pollution on UV exposure, it is reasonable to assume that air pollution can lead to myopia through the DA pathways affected by reduced UV exposure.

### Direct pathways

#### Peripheral hyperopia defocus

Peripheral hyperopia defocus indicates that peripheral images are focused behind the retinal surface, whereas the foveal image falls exactly on the retina (Rotolo et al. 2017). Animal experiments have proposed a mechanism in which the retina can “read” the direction of focus of incoming light rays on the retina and affect the choroid to actively change its thickness to move the retina towards the image plane via changes in retinal homeostasis mediated by neurotransmitters (Kubota et al. 2021). If the focal plane is behind the retina in areas of the visual field, axial growth is promoted owing to thinning of the subfoveal choroid (Schaeffel 2017; Kubota et al. 2021). Accumulating evidence has demonstrated that alterations of the focal plane can be related to ocular surface diseases, and the biophysical property changes of corneal cells involved in these diseases may reflect myopia progression, as the cornea contributes more than 60% of the focusing power (Xin et al. 2021).

Previous investigations have revealed a significant association between continuously increasing air pollution and ocular surface disorders, such as uveitis (Bai et al. 2021), keratitis (Sendra et al. 2021), and conjunctivitis (Antonini et al. 2021; Nucci et al. 2017). NO is converted to NO_2_, followed by the generation of O_3_ (Miyazaki et al. 2019). NO_2_ and O_3_ can directly damage the ocular surface by oxidation (Hong et al. 2016), acidification of tears, allergic sensitization, and chemical modification of aeroallergens (Lu et al. 2021). Allergic conjunctivitis (AC) is a common ocular surface disease that causes dry eye, itching, and burning sensations, and may lead to sight-threatening conditions (Lu et al. 2021). Reports from highly prevalent regions of AC suggest that air pollutants may be associated with increased sensitization (Miyazaki et al. 2020) and can induce or aggravate AC symptoms. Lu et al. (2021) found that the incidence of AC was positively correlated with PM_2.5_, PM_10_, CO, NO_2_, and O_3_. The number of outpatient visits with AC increased as the concentrations of NO_2_ (Hong et al. 2016; Wei et al. 2019; Miyazaki et al. 2020), O_3_ (Hong et al. 2016; Miyazaki et al. 2020), PM_2.5_ (Mu et al. 2020; Miyazaki et al. 2020), and PM_10_ (Miyazaki et al. 2020) changed. A correlation between allergic inflammation and subsequent myopia risk has also been established (Lin et al. 2016).

In patients with AC, immune-mediated destruction of the conjunctival epithelium is triggered when the ocular surface encounters environmental antigens. Subsequently, activated T cells and mast cells would specifically target sensitized ocular surface tissues, reduce mucin-secreting cells and break down integrity (Jung et al. 2018; Wei et al. 2018). Additionally, PM and NO_x_ have been suggested to alter the barrier integrity of the cornea due to the induced increase in ROS (Wei et al. 2019; Lasagni Vitar et al. 2019). Overloading of the antioxidative defense system in the conjunctiva leads to the production of inflammatory cytokines (Wolkoff 2017). Wei et al. (2018) verified that TNF-α and IL-6 reduced the levels of claudin-1 and zonula occludens-1 tight junction proteins in the barrier of corneal epithelial cells. Consequently, elevated inflammatory mediator release and corneal injury caused by AC have been proposed as possible mechanisms for increased corneal curvature. Wei et al. (2018) performed a cohort study and confirmed that children with AC had a 2.35-fold higher incidence of myopia than those without AC. They also observed that the rats with AC developed myopia. Additionally, the axial lengths of the eyes with AC were significantly longer than those of the control eyes (Wei et al. 2018). A case–control study conducted by Wang et al. (2021a) reported that patients with AC had an increased risk of keratoconus, which is mainly characterized by progressive corneal thinning and cone-shaped corneal protrusion (Wang et al. 2021a, 2021b; Ahmed et al. 2021). This implies a steeper central cornea and a flatter periphery. The relatively negative spherical aberration in the periphery causes peripheral hyperopia defocus and stimulates eyeball growth (Atchison and Rosén 2016), which, in turn, promotes the development of myopia.

#### Retinal ischemia

Vascular densities are reported to be significantly negatively correlated with axial length, including the capillaries in the superficial and deep macula, peripapillary area, and choroid (Wang et al. 2021c; Zheng et al. 2015; Liu et al. 2021). Quantitatively measured alterations in retinal vascular diameter may imply a retina abnormality (Chen et al. 2017). The decrease in microvascular density could conceivably lead to reduced metabolic demands in the local retinal region because it serves as a direct source of oxygen and nutrients for retinal pigment epithelium cells and retinal nerve fiber layer (RNFL). The hypoxic microenvironment has been shown to upregulate the expression of tissue hypoxia marker (Liu et al. 2021) and reduce the density of retinal pigment epithelium cells in the retro-equatorial region, causing a stretch of axial elongation (Wu et al. 2019). Grudzińska et al. (2022, Zheng et al. 2015) found a positive correlation between peak systolic velocity and end-diastolic velocity in the central retinal artery and mean thickness of the RNFL, ganglion cell, and inner plexus layer, and size of the rim area (the area located between the edge of the disc and the physiological cup containing the neural elements). Consequently, it can induce parapapillary RNFL thinning and impair retinal neuroactivity, which regulates the axial growth of eyes early in life. Additionally, special attention has been paid to the less tortuous retinal vessels, which can also cause hypoperfusion of the retina (Zheng et al. 2015).

Clinical investigations (Table 2) showed an inverse association between air pollution concentrations (measured as PM_2.5_, PM_10_, BC, O_3_, and NO_2_ concentrations) and central retinal arteriolar diameter, one of the first branches of the ophthalmic artery (Zhang et al. 2018; Baldoncini et al. 2019; La Spina et al. 2016). As for PM_2.5_, 4607 participants were examined in the analysis conducted by Adar et al. (2010), who found that central retinal artery diameter was negatively associated with increased long- and short-term levels of PM_2.5_. Provost et al. (2017) confirmed that each 10-µg/m^3^ increase in same-day exposure to PM_2.5_ was associated with 0.62 μm (95% CI: − 1.12, − 0.12) narrower retinal arterioles in school-aged children in Belgium. PM_10_ is a 2.5–10-μm-sized aerosol that contains coarse suspended materials, including pollen, fungal spores, and dust, which can serve as allergens or adjuvants (Miyazaki et al. 2020). Louwies et al. (2016, Louwies et al. 2013) suggested that PM_10_ exposure was associated with retinal arteriolar narrowing and venular widening. BC, a by-product of fuel combustion and one of the most toxic components of PM (Witters et al. 2021; Rabito et al. 2020), is associated with systemic inflammation and oxidative stress (Louwies et al. 2015). Louwies et al. (2013) indicated that there was a decrease in retinal artery diameter for each 1-µg/m^3^ increase in BC. They also observed a positive association between retinal venules and BC exposure (Louwies et al. 2015). Additionally, Korsiak et al. (2021) indicated that O_x_ (the combined oxidant capacity of O_3_ and NO_2_ using a redox-weighted average) was inversely associated with retinal arteriolar diameter, and the strongest association was observed for a 7-day mean exposure.

**Table 2:** Summary of clinical studies on the effects of ambient air pollutant exposure on retinal vessels

Study	Study period and location	Sample size	Age (years)	Environmental exposure measured	Outcome measured	Findings
Adar et al. (2010)	2002–2003, America	4607 adults	45–84	Long-term outdoor concentrations of PM_2.5_ estimated at each participant’s home using a spatio-temporal model	CRAE and CRVE	With − 0.8- and − 0.4-µm decreases in CRAE per interquartile increases in long- (3 µg/m^3^) and short-term (9 µg/m^3^) PM_2.5_ levels, respectively
Louwies et al. (2013)	January–May 2012, Belgium	84 adults	22–63	PM_10_ and BC levels measured at a nearby official monitoring station	CRAE and CRVE	Each 10-µg/m^3^ increase in PM_10_ associated with a 0.93-µm decrease in CRAE and a 0.86-µm decrease in CRVE Each 1-µg/m^3^ increase in BC associated with a 1.84-µm decrease in CRAE
Louwies et al. (2015)	1 week between April and May 2013, the north of Belgium	55 healthy nurses	22–59	Personal subchronic BC exposure measured continuously with a portable MicroAeth Model AE51	CRAE and CRVE	Increased exposure of 631-ng/m^3^ BC associated with 5.65-μm increase in CRAE
Louwies et al. (2016)	December 2014–April 2015, Flanders, Belgium	50 healthy adults	23–58	PM_10_ data measured at a nearby monitoring station	CRAE and CRVE	Each short-term increase of 10 µg/m^3^ PM_10_ during the 24 h preceding the study visit associated with a 0.58-µm decrease in CRAE, a 0.99-µm increase in CRVE
Provost et al. (2017)	2012–2014, Flanders, Belgium	221 children	8–12	Recent (same and previous day) and chronic (yearly mean) exposure modeled at the child’s residence using a high-resolution interpolation model	Retinal vessel diameters	Each 10-µg/m^3^ increment in same-day exposure to PM_2.5_ at school associated with 0.35-µm narrower retinal arterioles and 0.35-µm wider venules
Korsiak et al. (2021)	2018–2020, Vancouver Island, Canada	64 children	4–12	Daily mean outdoor PM_2.5_ concentrations measured by the provincial air monitoring station and Partisol 2025i sequential air sampler; O_3_ and NO_2_ measured at the provincial air monitoring site	CRAE and CRVE	O_x_ inversely associated with retinal arteriolar diameter The strongest association observed for 7-day mean exposures, where each 10-ppb increase in O_x_ associated with a 2.63-μm decrease in arteriolar diameter Weak inverse associations observed between PM_2.5_ and arteriolar diameter only at higher concentrations of O_x_

*PM*_*2.5*_ particulate matter 2.5, *PM*_*10*_ particulate matter 10, *BC* black carbon, *O*_*3*_ ozone, *NO*_*2*_ nitrogen dioxide, *O*_*x*_ the combined oxidant capacity of O_3_ and NO_2_ using a redox-weighted average, *CRAE* central retinal artery equivalent, *CRVE* central retinal vein equivalent.

The biological mechanisms underlying the impact of air pollution on the retinal microvasculature are thought to be related to inflammation and oxidative stress pathways. We propose two possible explanations for this finding.

One mechanism is that systemic microvascular endothelium-dependent dilation affected by pulmonary air pollutant exposure, particularly PM, can be related to changes in retinal blood vessels, as they share great similarities in development and anatomy with the microvasculature of the heart, lungs, and brain (Louwies et al. 2013). There is rapidly developing evidence supporting the role of exposure to air pollution in pulmonary inflammation and subsequent low-grade, systemic inflammation (Marchini et al. 2020; Gao et al. 2020; Li et al. 2019). Louwies et al. (2016) suggested a possible role for PM_10_ in downregulating the expression of microRNAs extracted from venous blood. Differential regulation of microRNAs involved in oxidative stress and inflammatory processes may have contributed to arteriolar narrowing and venular widening. Animals exposed to different types of particles exhibit thickening of alveolar walls, neutrophil recruitment, and macrophage activation, thus increasing systemic oxidative stress and inflammation response, as well as stimulating the generation of ROS and pro-inflammatory cytokines, which finally enter circulation (de Souza Xavier Costa et al. 2020). The substances involved in these systemic reactions may take some time to affect the reactivity of retinal blood vessels, even several hours after exposure, according to Louwies et al. (2013). For instance, ROS that flows to the retina in the bloodstream leads to endothelial nitric oxide synthase uncoupling and reduces the bioavailability of the vasodilator NO, which contributes to endothelial dysfunction and vasoconstriction (Korsiak et al. 2021; Louwies et al. 2013). These alterations in retinal microcirculation, including arteriolar damage and endothelial dysfunction, create a situation of diminished blood flow and impair retinal neuroactivity consequently (Dadvand et al. 2017; De Boever et al. 2014). In turn, it is a source of chronic oxidative stress and inflammation (Chan et al. 2020).

Another hypothesis is that air pollutants can directly affect ocular development and retinal activity owing to their water solubility, concentration, and ability to oxidize tissues. Ultrafine PMs that can pass through the capillary membrane are readily picked up by cells and induce cellular damage (Schikowski 2022). CO emitted from diesel engine exhaust fumes and traffic congestion (Supharakonsakun et al. 2020) is highly soluble, non-irritating, and readily passes through the bloodstream. The toxicity of CO results from its successful competition with oxygen in binding with hemoglobin, decreasing blood oxygen delivery, and resulting in acute tissue hypoxia (Schikowski 2022; Bertrand et al. 2020), whereas NO also attaches to the hemoglobin and other iron-containing proteins because of its binding affinity (Schikowski 2022). Moreover, as gaso-transmitters in vivo, CO and NO transgress cells and tissues rapidly and react with reactive chemical species causing abnormal biochemical reactions (Mahan 2020). However, whether air pollutants directly participate in hypoxic vasoconstriction requires further investigation.

Additionally, air pollution exposure may trigger an autonomic imbalance that favors a sympathetic response to the smooth muscles surrounding the blood vessels and affects retinal vascular densities (Korsiak et al. 2021; Koch et al. 2020). However, it is still controversial because it does not consider that retinal blood vessels lack functional sympathetic innervations (Louwies et al. 2013).

#### Other evidence linking air pollution to myopia

Other evidence, in addition to the current studies, suggests that the inflammatory response caused by long-term exposure to air pollution induces local biochemical reactions, resulting in direct tissue remodeling and progression of myopia. For example, matrix metalloproteinase-2 (MMP-2) is a scleral extracellular matrix degradation enzyme, and its activation induces collagen fiber I degradation, followed by loss of scleral connective tissue, together with scleral thinning and weakening, which leads to axial elongation and myopia (Ikeda et al. 2020; Lin et al. 2016). Inflammatory cytokines IL-6 and TNF-α in the retina may serve as triggers to initiate MMP-2 activity in the retina, followed by sclera (Yuan et al. 2019), causing progressive scleral remodeling and myopia. Moreover, resveratrol, a naturally occurring antioxidant, has been shown to ameliorate myopia development by blocking the relevant signaling pathways of inflammatory effects above (Hsu et al. 2021; Jiang et al. 2019; Wei et al. 2019).

## Conclusion

**Fig. 2:**
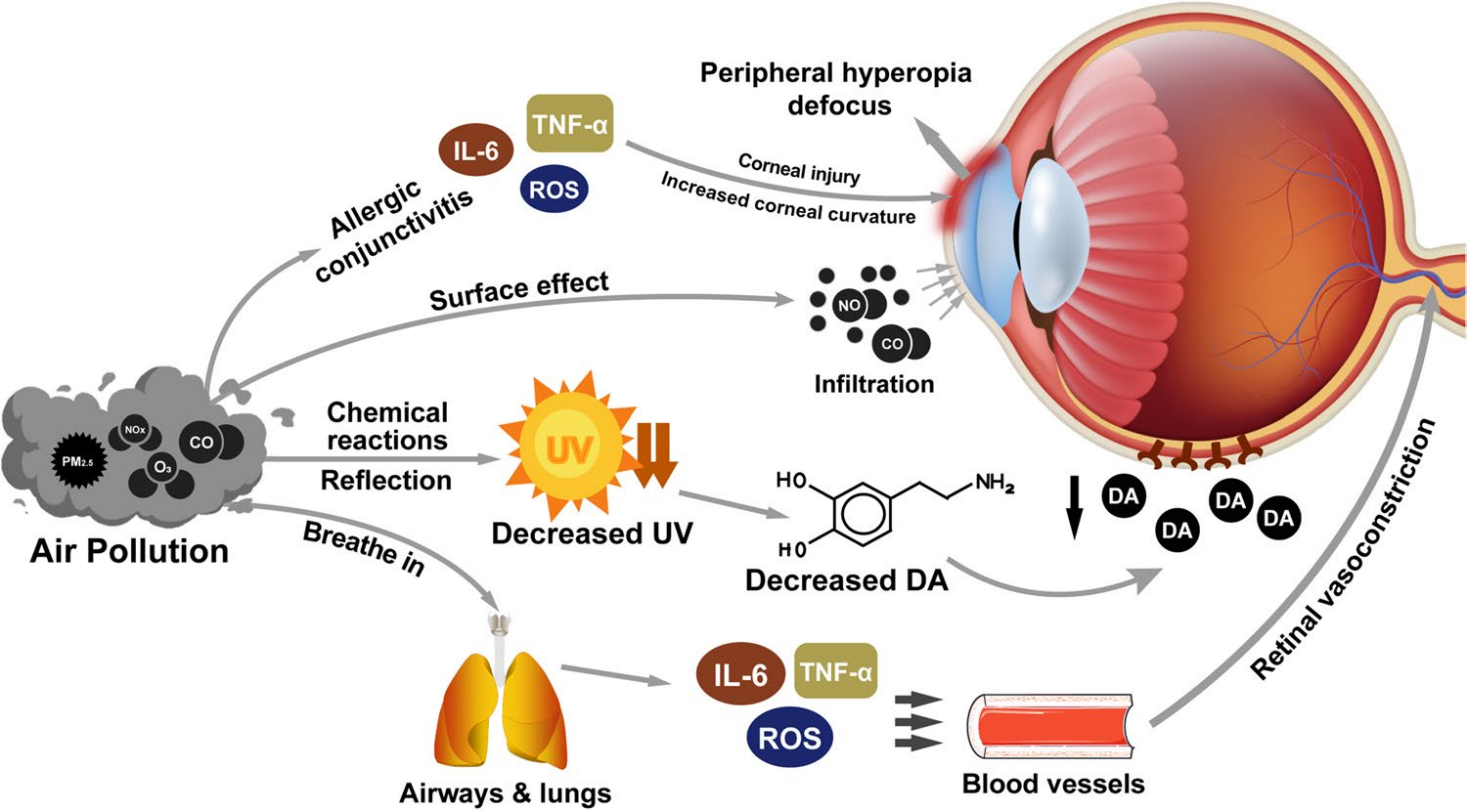
Potential mechanisms linking air pollution to myopia. Ambient air pollution may aggravate allergic conjunctivitis symptoms and cause corneal injury, which lead to peripheral hyperopia defocus and stimulate eyeball growth. The synthesis and emission of air pollutants lead to the reduction in ultraviolet exposure and retinal dopamine release. The pulmonary inflammatory factors and reactive oxygen species induced by air pollution can enter the blood circulation, resulting in systemic inflammation and oxidative stress, thus causing retinal ischemia and myopia. Furthermore, several air pollutants may directly induce hypoperfusion of the retina through ocular surface. *PM*_*2.5*_ particulate matter 2.5, *CO* carbon monoxide, *NO*_*x*_ nitrogen oxides, *NO* nitric oxide, *O*_*3*_ ozone, *IL-6* interleukin-6, *TNF-α* tumor necrosis factor-α, *ROS* reactive oxygen species, *UV* ultraviolet, *DA* dopamine

Exposure to ambient air pollution has a significant impact on the development of myopia. Although peripheral hyperopia defocus, the DA pathway, and retinal ischemia are all suggested to play a role (Fig. 2), the detailed mechanisms by which air pollutants interfere with myopia remain unclear and require further investigation. As most of the current epidemiological evidence is cross-sectional in nature, longitudinal studies that comprise a larger scale and quantified measurement of air pollution exposure are required in the future to elucidate the exact impact and provide proof of causality.

As aforementioned, air quality is far worse in Asia, and the severity of air pollution may have a substantial role in the etiology of myopia in these countries. Therefore, efforts to address air pollution are required to prevent the incidence and progression of myopia. For policymakers, methods such as establishing and strictly enforcing air quality standards and adopting policies against heavily polluting industries are advised. For the public, it is critical to raise awareness and limit individual exposure to preventable risks such as taking public transportation more.

## Data Availability

Data sharing is not applicable to this article as no new data were created or analyzed in this study.
